# Outdoor activities and social support on anxiety in Chinese older adults: a cross-sectional study

**DOI:** 10.3389/fpubh.2026.1817922

**Published:** 2026-05-18

**Authors:** Zhonghua Li, Honglin Dai, Ya Zhao

**Affiliations:** 1 College of Physical Education, Chengdu University, Chengdu, China; 2 School of Nursing, Chengdu University, Chengdu, China

**Keywords:** anxiety, outdoor activities, social support, older adult, mediation effect

## Abstract

**Objective:**

This study examined the associations between different types of outdoor activity and anxiety in older Chinese adults, and assessed whether social support was related to these associations as a potential mediator.

**Methods:**

Data were obtained from 8,813 adults aged 60 years and older who participated in the 2018 wave of the Chinese Longitudinal Healthy Longevity Survey. Outdoor activity was classified as Tai Chi, square dancing, neighborhood walking, other outdoor activities, and overall outdoor activity frequency. Anxiety symptoms were measured using the Generalized Anxiety Disorder-7 scale. Social support was assessed using a composite indicator covering family, caregiving, financial, and community-based support resources. Mediation analyses were conducted with 5,000 bootstrap resamples after adjustment for demographic, behavioral, residential, relational, and socioeconomic covariates.

**Results:**

Anxiety was identified in 11.57% of participants. Social support was consistently associated with lower anxiety scores. Overall outdoor activity, neighborhood walking, and other outdoor activities were associated with lower anxiety, and small indirect effects through social support were observed for these activity types. No significant mediation through social support was found for Tai Chi or square dancing. The observed correlations and regression coefficients were modest in magnitude.

**Conclusion:**

Routine outdoor activities, particularly neighborhood walking, were associated with lower anxiety levels in older adults, with social support accounting for part of these associations. Although the effect sizes were small and causal inferences cannot be drawn from the cross-sectional design, the findings suggest that accessible outdoor activities and supportive social environments may be relevant to healthy aging and later-life mental wellbeing.

## 1 Introduction

Anxiety disorders are among one of the most common mental diseases in all over the world. The prevalence rate for anxiety disorders is around 16.50% in old people (1) which are significantly correlated with functional impairments, social withdrawal, and low quality of life (2). With aging there is a tendency for older adults to be burdened by the mental stress caused by several comorbidities, pain, physical and impaired functioning. The same period is also marked by the frequent coexistence of anxiety with other mental disorders (3). For example, older adults who have existential worries or are concerned with the meaning of life and feel a sense of futility can be more susceptible to depression (4). Medications and psychotherapy are still used widely in the clinic. There are many treatments available to treat the symptoms of anxiety; however, there are some obstacles such as high costs of treatment, unwanted side effects of medicines, and lack of access to expert guidance that has limited their wider adoption (5). Thus, recent guidelines are encouraging the use of non-pharmaceutical interventions such as physical activity, dietary modifications, proper sleeping, interpersonal relations -as significant auxiliary strategies in treating anxiety amongst old-age folks (6–8). The anxiety disorders include the following categories: Social Phobia (SAD), Agoraphobia (AG), Specific Phobias (SP), generalized anxiety disorder (GAD), panic disorder (PD). Although they share some specific features at diagnosis, they have similar underlying maintenance mechanisms, in particular evasive behaviors and similar effects upon the performance of daily life activities (9, 10).

Previous studies suggest that regular physical activity is associated with lower anxiety levels (11). Longitudinal studies as well as meta-analyses have shown that physical activity at a moderate-to-high intensity level is significantly associated with lower risk for developing an anxiety disorder during adulthood, with these positive impacts especially seen in the aged population (12). In terms of older adults, it seems as if the environment in which one engages in physical activity can have an impact on its mental health effects; some evidence indicates that being active outdoors in a more natural setting could improve wellbeing via pathways such as reducing perceived stress or improving adherence due to enjoyment though studies on its effectiveness in comparison with the traditional indoor gym setting have mixed results (13, 14). Indicates thatindividuals who spend at least about 120 min per week in natural environments such as parks and green spaces report significantly better mental health and life satisfaction than those with lower exposure (15). Outdoor activity may be linked to anxiety through multiple pathways. Such as influence physiological regulatory systems, including the hypothalamic-pituitary-adrenal axis, the Monoamine System, and the Opioid System; these systems are closely linked to stress reactivity and to the manifestation of affective symptoms such as anxiety and depression (16), At the same time, outdoor settings provide nature-related exposures, including green space, sunlight, microbial contact, and aesthetics, which can offer additional protection for mental health (17–20). Thus, outdoor activity that combines physical activity with exposure to natural environments represents a compound health behavior. Its potential protective effects on anxiety likely arise from the additive contributions of multiple pathways. Because it generally requires less equipment, fewer venue constraints, and less professional support, outdoor activity may offer high accessibility and cost-effectiveness for public health and community interventions.

A large body of research indicates that social support is an important resource that buffers stress in older adults, fosters resilience, and mitigates loneliness and psychological distress (21, 22), Social support typically includes emotional support, instrumental support, and informational support (23). Emotional support can facilitate emotion regulation by providing empathy, understanding, and a sense of safety. Instrumental support can reduce situational demands, thereby reducing arousal, and it can buffer psychological distress by conveying that one is valued and by strengthening self-esteem and a sense of control. Insufficient or vague informational support can also lead to misunderstanding, fear, conflict, and anxiety (24). All three forms of support show a consistent association with lower levels of anxiety and psychological distress (25), whereas a lack of social relationships is strongly associated with increased risk of anxiety disorders (26). In addition, stable social ties are linked to reduced cortisol reactivity and diminished activity in the dorsal anterior cingulate cortex and Brodmann's area (27), providing neurophysiological evidence for the anxiety-buffering effects of social support.

Evidence linking physical activity and social support to better mental health in older adults is already substantial (28, 29), yet the mechanisms connecting them remain less clearly specified. Findings from large national samples, particularly among older adults in China, remain comparatively limited, and existing studies have largely remained at the correlational level, with insufficient systematic examination of the mediating role of social support. Much of the epidemiologic literature treats physical activity as a broad exposure, while giving less attention to routine outdoor activities that unfold in community settings and may carry distinct social meanings. This question becomes more salient in later life, when daily activity spaces tend to narrow and nearby environments increasingly shape both mobility and opportunities for social contact (30, 31). Against this background, it is not enough to ask whether older adults are active. What also matters is what outdoor activity can be embedded in everyday neighborhood life, and whether that social context helps explain variation in anxiety.

The present study was therefore guided by an integrated framework combining the ecological perspective on later-life behavior with the stress-buffering model of social support. From the ecological perspective, outdoor activity in later life is shaped not only by individual capacity, but also by the environmental conditions of nearby communities, especially those related to mobility, accessibility, and opportunities for everyday social contact (29). The stress-buffering perspective adds another layer to this pathway: social support may be related to mental health directly, while also reducing the adverse psychological effects of stress on anxiety (32). Longitudinal evidence further shows that weaker social connectedness can heighten perceived isolation, which in turn predicts subsequent anxiety symptoms in older adults (26). Based on these perspectives, the present study adopted a conceptual model in which outdoor activity may be associated with anxiety both directly and indirectly through social support. Clarifying whether routine outdoor activities embedded in neighborhood life are associated with anxiety, and whether social support helps explain that association, has clear public health relevance. A better understanding of these pathways could inform community-based strategies for healthy aging by identifying feasible forms of outdoor activity for older adults and the social conditions under which such activities may be associated with anxiety in later life.

## 2 Method

### 2.1 Study participants

Data were drawn from the 2018 cross-sectional wave of the Chinese Longitudinal Healthy Longevity Survey (CLHLS). The CLHLS is organized and implemented by the Center for Healthy Aging and Development Studies / National School of Development at Peking University. It is a large-scale, national, and comprehensive longitudinal social survey of older adults in China. The study protocol was approved by the Biomedical Ethics Committee of Peking University (IRB00001052-13074). The survey uses a multistage, stratified, and disproportionate sampling design covering 23 provinces, municipalities, and autonomous regions, and the sample has good representativeness. A total of 15,874 adults aged 60 years and older participated in the 2017–2018 survey. All participants took part voluntarily and provided written informed consent.

**Figure 1:**
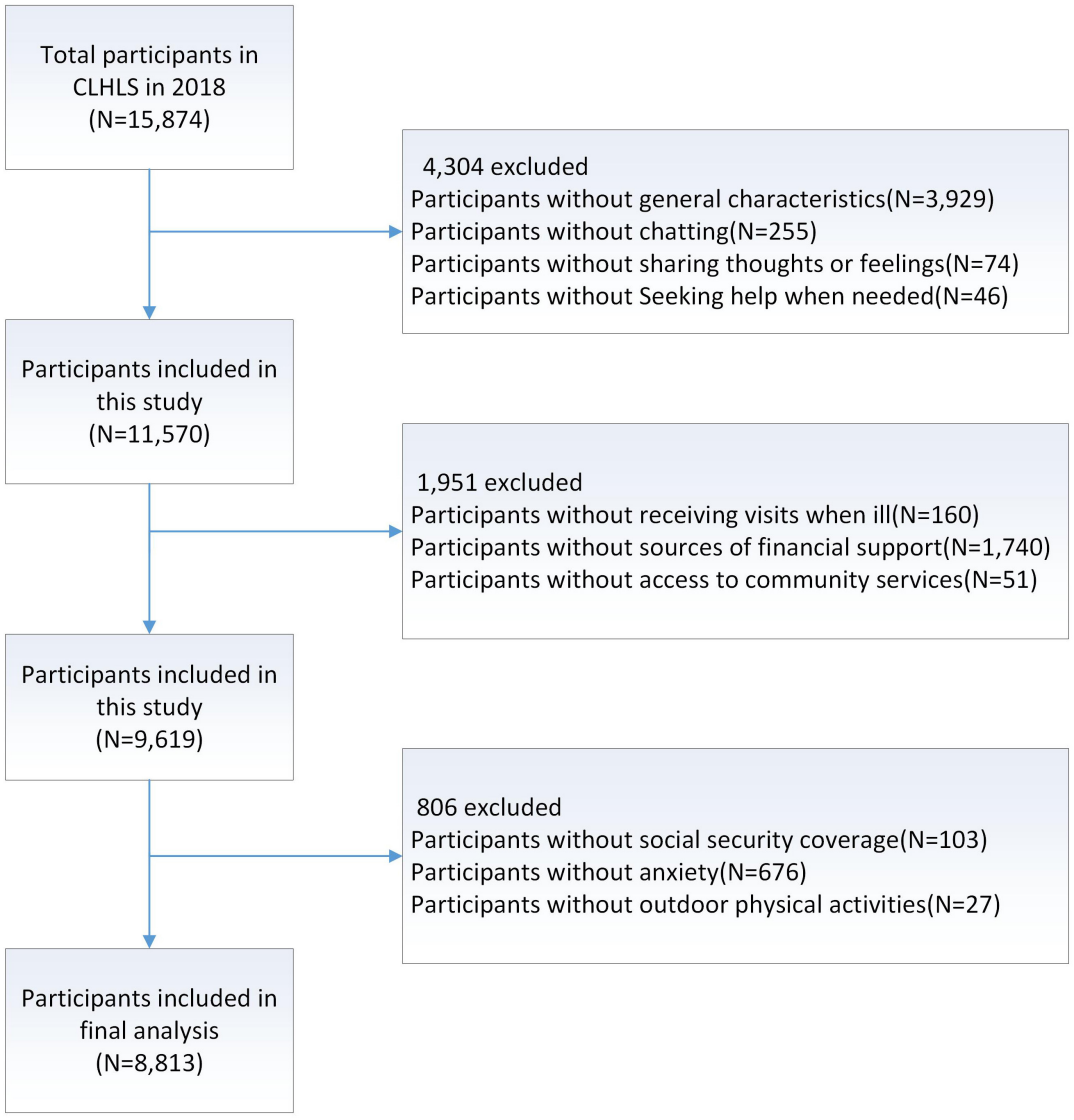
Flow diagram of participant selection.

Based on the study aims, we included: (1) participants aged 60 years and older who completed the outdoor activity module, the anxiety assessment, and the social support assessment; and (2) participants from the 2017–2018 survey waves with complete data on health-related lifestyle measures. Exclusion criteria were: (1) participants lost to follow-up during the study period; (2) participants who did not complete the relevant scales in the 2017–2018 survey; and (3) after applying these criteria, a final analytic sample of 8,813 participants was retained. The participant selection flow is shown in Figure 1.

### 2.2 Measurement metrics

#### 2.2.1 Outdoor activities

Based on CLHLS questionnaire items on older adults' participation frequency in Tai Chi, square dancing, neighborhood walking, and other outdoor activities, we recoded the original frequency scores (1 = does not participate, 2 = less than monthly but sometimes, 3 = less than weekly but at least once per month, 4 = less than daily but at least once per week, 5 = almost every day) into three categories: never (1), sometimes (2, 3), and often (4, 5). Overall outdoor activity was then defined as follows: participants were classified as “often” if any of the four activities had a score ≥4, or if all four activities scored 3; classified as “never” if all four activities scored 1; and all other combinations were classified as “sometimes” (33).

#### 2.2.2 Anxiety

Anxiety symptoms were assessed by the Generalized Anxiety Disorder scale with 7 items (GAD-7), which asked how often they occurred during the previous two weeks on a list of symptoms that are commonly associated with anxiety disorders; scores per item ranged between 0 and 3, with0 = not at all 1 = some days; 2 = more than half the days; 3 = all days. Total range: 0–21. Participants score of 5′and above in the scale were considered to have anxiety. This was found to be internally consistent (Cronbach's alpha = 0.917).

#### 2.2.3 Social support

Social support was defined as a multidimensional construct that encompassed both functional and structural aspects of support. Functional support referred to the supportive functions received by older adults, including everyday communication, emotional disclosure, help-seeking in times of difficulty, illness-related caregiving, and financial assistance from adult children. Structural support referred to the availability of supportive ties and broader social resources, including frequent visits from children, access to community-based social services, and participation in social insurance programs. Together, these indicators reflected the overall level of support available from family, informal social networks, community services, and the social welfare system (34, 35).

Functional Support. Three questions were used that asked about respondents' social participation activities, as well as their primary source(s) of assistance: “Who do you talk to most often on a day-to-day basis?”(2) “With whom would you most like to speak if you wanted to discuss something about yourself, either a thought or an emotion,” and (3)“When you encounter problems or confusion, who do you usually ask for help?” Respondents identified their usual sources of support from a predefined list that included a spouse, children, in-laws, grandchildren, other relatives, friends, neighbors, social workers, caregivers (e.g., a nanny), as well as the options “no one” and “online friends.” To construct an index that captured both the breadth and intensity of available support, we applied a weighted scoring scheme in which support from a spouse was assigned 3 points; support from immediate family members, other relatives, friends, or neighbors was assigned 2 points; and support from formal providers such as social workers or paid caregivers was assigned 1 point. Reporting no available supporter was coded as 0, so higher values reflected stronger perceived support. Illness-related caregiving support was assessed with the question “When you are sick, who usually provides care for you?,” using the same response categories and scoring rules as functional support (range 0–3). Financial assistance from adult children was measured with three items asking, respectively, “In the past year, how much cash or in-kind support did you receive from your son or daughter-in-law?,” “...from your daughter or son-in-law?”, and “...from any grandchild?” Any reported cash or in-kind support was coded as 1, and none as 0. For children's visits, basic information about children was collected and participants were asked whether they had children who visited frequently (yes/no); yes was coded as 1 and no as 0. External social support was assessed by quantifying access to social services and participation in social insurance programs. Social services included personal care, home medical services, psychological counseling, assistance with daily shopping, social and recreational activities, legal aid, health education, and community dispute mediation. Social insurance participation included retirement wages or pensions, private pension insurance, public free medical services, cooperative medical schemes, basic medical insurance, critical illness insurance, and life insurance. If any item in social services or social insurance was answered “yes,” the participant was considered to have access to community services or social insurance (coded as 1); otherwise, coded as 0.

The composite social support score was calculated by summing the six subdimensions, resulting in a comprehensive index of social support resources. Scores ranged from 0 to 16, with higher scores indicating broader social support.

#### 2.2.4 Covariate

The analysis included demographics, behavior, and economic status as covariates for the regression model. Demographics included gender (female/male),ethnicity (Han Chinese vs. ethnic minority), and age group (<70 years old, 70 to 79 years old, 80 to 89 years old and ≥90 years old).The health behavior variables considered are smoking, drinking, and having at least one chronic disease vs. no chronic disease. The residential and relational status covariates include residence type (urban, residence (urban area, city, or rural community), marital status (marital union or not in a union). Socioeconomic status: determined by highest completed schooling and annual family income. Education level was categorized as elementary education or lower, middle school, and technical college or higher. Family income was divided into four quartiles: Q1 (<25,000 yuan), Q2 (25,000–49,999 yuan), Q3 (50,000–74,999 yuan), and Q4 (≥75,000 yuan).These covariates were simultaneously adjusted for in all mediation models.

### 2.3 Statistical analysis

We performed independent sample *t*-test or ANOVA to compare the difference on the level of social support among different subgroups; and Chi-square test for difference in the susceptibility rate of anxiety among different subgroups. Correlations were examined between the frequency of exercising outside. Spearman's rank correlation was used to examine the relationships between anxiety symptoms and perceived social support.

A simple mediation model was fitted separately for each outdoor activity variable, with outdoor activity as the independent variable(X), social support as the mediator(M), and anxiety symptom score as the dependent variable(Y), The indirect effect was defined as a × b, the direct effect as c′, and the total effect as c, while adjusting for demographics, behavior, residential and relational status, and socioeconomic status; indirect effects were estimated using 5,000 bootstrap resamples. Subgroup mediation analyses were further performed by sex, age group, chronic disease status, and residence type using the same analytic framework and bootstrap procedure as in the main models. Given the limited stability of some subgroup estimates, these results were interpreted descriptively. Subgroup mediation analyses were limited to overall outdoor activity, neighborhood walking, and other outdoor activity. Tai Chi and square dancing were not included because their participation rates were very low, and further stratification produced sparse and highly unbalanced subgroup distributions, making the mediation estimates insufficiently stable for reliable interpretation. We conducted statistical analyses with Stata MP 18software (*p* < 0.05) for all two-sided tests.

## 3 Result

A total of 8,813 participants were included. Women accounted for 56.01% of the sample, and most participants were Han ethnicity (94.54%). The overall age structure was highly aged, with participants aged ≥90 years accounting for 38.47%, whereas those aged <70 years accounted for only 12.0%. Most participants lived in rural (43.21%) or towns (34.04%). Educational attainment was generally low, with 81.7% having primary school education or below. More than half had no spouse/partner (57.98%). The prevalence of chronic conditions was high (72.26%), while the proportions reporting smoking (15.43%) and alcohol use (14.51%) were relatively low. Regarding outdoor activity, frequent Tai Chi and square dancing were reported by only 2.1% and 3.74%, respectively. In contrast, neighborhood walking was common, with 43.37% reporting frequent participation. Overall, 52.75% reported frequent outdoor activity, whereas 34.52% reported never participating in outdoor activities. See Table 1.

**Table 1:** Analyses of social support scores across different demographic characteristics.

Variable	Category	Number (n)	Social support scores (Mean ±SD)	t/F	*P*
Sex	Male	3,877	12.84 ± 2.29	*t* = 20.51	**<0.001** ^***^
	Female	4,936	11.89 ± 2.07		
Ethnicity	Han	8,332	12.32 ± 2.23	*t* = 1.66	0.097
	Ethnic minority	481	12.15 ± 2.07		
Smoking	Yes	1,360	12.81 ± 2.30	*t* = 9.04	**<0.001** ^***^
	No	7,453	12.22 ± 2.19		
Alcohol consumption	Yes	1,279	12.89 ± 2.26	*t* = 10.20	**<0.001** ^***^
	No	7,534	12.21 ± 2.20		
Chronic conditions	Yes	6,368	12.38 ± 2.24	*t* = 4.91	**<0.001** ^***^
	No	2,445	12.12 ± 2.15		
Marital status	Without spouse	5,110	10.93 ± 1.42	*t* = −99.79	**<0.001** ^***^
	With spouse	3,703	14.21 ± 1.64		
Age^1^	<70 years	1,054	13.73 ± 2.12	*F* = 655.85	**<0.001** ^***^
	70–80 years	2,233	13.28 ± 2.22		
	80–90 years	2,136	12.25 ± 2.17		
	≥90 years	3,390	11.27 ± 1.68		
Place of residence^2^	city	2,005	12.16 ± 2.33	*F* = 7.80	**<0.001** ^***^
	Town	3,000	12.29 ± 2.22		
	Rural	3,808	12.40 ± 2.14		
Educational level3	Primary school or below	7,197	12.15 ± 2.14	*F* = 101.92	**<0.001** ^***^
	Secondary school	1,350	13.06 ± 2.39		
	College or above	266	12.68 ± 2.49		
Income quartile^4^	Q1	3,676	12.27 ± 2.29	*F* = 2.63	**0.048** ^*^
	Q2	1,341	12.38 ± 2.18		
	Q3	1,199	12.44 ± 2.06		
	Q4	2,597	12.27 ± 2.20		
Tai Chi^5^	Never	8,568	12.29 ± 2.21	*F* = 6.10	**0.002** ^**^
	Sometimes	60	12.77 ± 2.68		
	Frequent	185	12.81 ± 2.27		
Square dancing^6^	Never	8,370	12.28 ± 2.22	*F* = 11.34	**<0.001** ^***^
	Sometimes	114	12.79 ± 2.29		
	Frequent	329	12.80 ± 2.16		
Neighborhood walking^7^	Never	3,718	11.79 ± 2.17	*F* = 184.30	**<0.001** ^***^
	Sometimes	1,272	12.68 ± 2.21		
	Frequent	3,823	12.69 ± 2.17		
Other outdoor activities^8^	Never	5,957	12.10 ± 2.18	*F* = 86.72	**<0.001** ^***^
	Sometimes	722	12.75 ± 2.30		
	Frequent	2,134	12.75 ± 2.21		
Overall outdoor activity^9^	Never	3,042	11.67 ± 2.11	*F* = 201.72	**<0.001** ^***^
	Sometimes	1,122	12.60 ± 2.22		
	Frequent	4,649	12.66 ± 2.19		

^***^p < 0.001, ^**^p < 0.01, ^*^p < 0.05. Bonferroni correction was applied for all post hoc comparisons. ^1^Pairwise comparisons among the four age groups were all significant (all *p* < 0.001).^2^Rural > Urban (^***^); Town vs. Urban and Rural vs. Town were not significant (*p* > 0.05). 3Secondary school > Primary school or below (^***^); College or above > Primary school or below (^***^); Secondary school > College or above (^*^). ^4^Overall difference p = 0.048; after Bonferroni correction, no pairwise comparisons were significant (*p* > 0.05). ^5^Frequent > Never (^**^); Sometimes > Never (^**^); Frequent vs. Sometimes not significant (*p* > 0.05). ^6^Sometimes > Never (^**^); Frequent > Never (^**^); Sometimes vs. Frequent not significant (*p* > 0.05). ^7^Sometimes > Never (^**^); Frequent > Never (^**^); Sometimes vs. Frequent not significant (*p* > 0.05). ^8^Sometimes > Never (^**^); Frequent > Never (^**^); Sometimes vs. Frequent not significant (*p* > 0.05). ^9^Sometimes > Never (^**^); Frequent > Never (^**^); Sometimes vs. Frequent not significant (*p* > 0.05).

Between-group comparisons showed significant differences in social support scores by sex, smoking status, alcohol use, chronic condition status, marital status, age group, residence type, education level, and income (*p* < 0.05). Higher social support scores were observed among men, smokers, drinkers, participants with chronic conditions, those with a spouse/partner, younger age groups, rural residents, and those with secondary education. In terms of behavioral characteristics, participation levels in Tai Chi, square dancing, neighborhood walking, other outdoor activities, and overall outdoor activity were all significantly associated with social support (*p* < 0.05). Participants who engaged in outdoor activities had higher social support scores than those who never participated. See Table 1.

Chi-square tests indicated that 1,020 participants (11.57%) exhibited anxiety. The prevalence of anxiety was higher among women, participants with chronic conditions, town/rural residents, those with primary school education or below, and lower-income groups (*p* < 0.001). In contrast, smokers (*p* = 0.003) and drinkers (*p* < 0.001) had a lower of anxiety. Among outdoor activity factors, neighborhood walking (*p* < 0.001), other outdoor activities (*p* = 0.007), and overall outdoor activity frequency (*p* < 0.001) were significantly associated with anxiety. See Table 2.

**Table 2:** Prevalence of anxiety by participant characteristics.

Variable	Category	Total, *n* (%)	No anxiety, *n* (%)	Anxiety, *n* (%)	χ^2^	P
Sex	Male	3,877 (43.99)	3,534 (91.15)	343 (8.85)	**50.2901**	**<0.001** ^***^
	Female	4,936 (56.01)	4,259 (86.28)	677 (13.72)		
Ethnicity	Han	8,332 (94.54)	7,359 (88.32)	973 (11.68)	1.6152	0.204
	Ethnic minority	481 (5.46)	434 (90.23)	47 (9.77)		
Age	<70 years	1,054 (11.96)	913 (86.62)	141 (13.38)	6.8328	0.077
	70– <80 years	2,233 (25.34)	1,965 (88.00)	268 (12.00)		
	80– <90 years	2,136 (24.24)	1,885 (88.25)	251 (11.75)		
	≥90 years	3,390 (38.47)	3,030 (89.38)	360 (10.62)		
Smoking status	Yes	1,360 (15.43)	1,235 (90.81)	125 (9.19)	**8.9205**	**0.003** ^**^
	No	7,453 (84.57)	6,558 (87.99)	895 (12.01)		
Alcohol consumption	Yes	1,279 (14.51)	1,182 (92.42)	97 (7.58)	**23.2705**	**<0.001** ^***^
	No	7,534 (85.49)	6,611 (87.75)	923 (12.25)		
Chronic conditions	Yes	6,368 (72.26)	5,564 (87.37)	804 (12.63)	**24.8125**	**<0.001** ^***^
	No	2,445 (27.74)	2,229 (91.17)	216 (8.83)		
Place of residence	city	2,005 (22.75)	1,843 (91.92)	162 (8.08)	**34.5754**	**<0.001** ^***^
	Town	3,000 (34.04)	2,597 (86.57)	403 (13.43)		
	Rural	3,808 (43.21)	3,353 (88.05)	455 (11.95)		
Marital status	Without spouse	5,110 (57.98)	4,499 (88.04)	611 (11.96)	1.7444	0.187
	With spouse	3,703 (42.02)	3,294 (88.95)	409 (11.05)		
Educational level	Primary school or below	7,197 (81.66)	6,297 (87.49)	900 (12.51)	**33.2723**	**<0.001** ^***^
	Secondary school	1,350 (15.32)	1,250 (92.59)	100 (7.41)		
	College or above	266 (3.02)	246 (92.48)	20 (7.52)		
Annual household income	Q1	3,676 (41.71)	3,147 (85.61)	529 (14.39)	**49.9204**	**<0.001** ^***^
	Q2	1,341 (15.22)	1,203 (89.71)	138 (10.29)		
	Q3	1,199 (13.60)	1,085 (90.49)	114 (9.51)		
	Q4	2,597 (29.47)	2,358 (90.80)	239 (9.20)		
Tai Chi	Never	8,568 (97.24)	7,576 (88.42)	992 (11.58)	0.9955	0.608
	Sometimes	60 (0.68)	51 (85.00)	9 (15.00)		
	Frequent	185 (2.10)	166 (89.73)	19 (10.27)		
Square dancing	Never	8,370 (94.99)	7,393 (88.33)	977 (11.67)	4.3993	0.111
	Sometimes	114 (1.29)	98 (85.96)	16 (14.04)		
	Frequent	329 (3.74)	302 (91.79)	27 (8.21)		
Neighborhood walking	Never	3,718 (42.20)	3,248 (87.36)	470 (12.64)	**15.5855**	**<0.001** ^**^
	Sometimes	1,272 (14.44)	1,106 (86.95)	166 (13.05)		
	Frequent	3,823 (43.37)	3,439 (89.96)	384 (10.04)		
Other outdoor activities	Never	5,957 (67.59)	5,240 (87.96)	717 (12.04)	**9.974**	**0.007** ^**^
	Sometimes	722 (8.19)	627 (86.84)	95 (13.16)		
	Frequent	2,134 (24.22)	1,926 (90.25)	208 (9.75)		
Overall outdoor activity	Never	3,042 (34.52)	2,641 (86.82)	401 (13.18)	**24.7232**	**<0.001** ^***^
	Sometimes	1,122 (12.73)	967 (86.19)	155 (13.81)		
	Frequent	4,649 (52.75)	4,185 (90.02)	464 (9.98)		

^***^p < 0.001, ^**^p < 0.01, ^*^p < 0.05.

Correlation analyses showed positive correlations among the outdoor activity types (*p* < 0.001). Social support was weak positive correlation correlated with overall outdoor activity frequency (*r* = 0.206, *p* < 0.001) and with Tai Chi (*r* = 0.041, *p* < 0.001), square dancing (*r* = 0.049, *p* < 0.001), neighborhood walking (*r* = 0.198, *p* < 0.001), and other outdoor activities (*r* = 0.143, *p* < 0.001). Social support was weaklynegatively correlated with anxiety (*r* = −0.035, *p* < 0.001). Overall outdoor activity frequency was weaklynegatively correlated with anxiety (*r* = −0.024, *p* < 0.05), whereas correlations between Tai Chi, square dancing, neighborhood walking and other outdoor activities and anxiety were not significant. These findings indicate that the bivariate associations among outdoor activity, social support, and anxiety were generally limited in magnitude. See Table 3.

**Table 3:** Correlations among outdoor activities, social support, and anxiety.

Variable	Tai Chi	Square dancing	Neighborhood walking	Other outdoor activities	Overall outdoor activity	Social support	Anxiety
Tai Chi	1						
Square dancing	**0.289** ^***^	1					
Neighborhood walking	**0.060** ^***^	**0.059** ^***^	1				
Other outdoor activities	**0.137** ^***^	**0.082** ^***^	**0.311** ^***^	1			
Overall outdoor activity	**0.132** ^***^	**0.175** ^***^	**0.841** ^***^	**0.529** ^***^	1		
Social support	**0.041** ^***^	**0.049** ^***^	**0.198** ^***^	**0.143** ^***^	**0.206** ^***^	1	
Anxiety	0.007	−0.002	−0.01	−0.014	**−0.024** ^***^	**−0.035** ^***^	1

Correlations are Spearman's rank correlation coefficients. ^***^p < 0.001, ^**^p < 0.01, ^*^p < 0.05.

**Figure 2:**
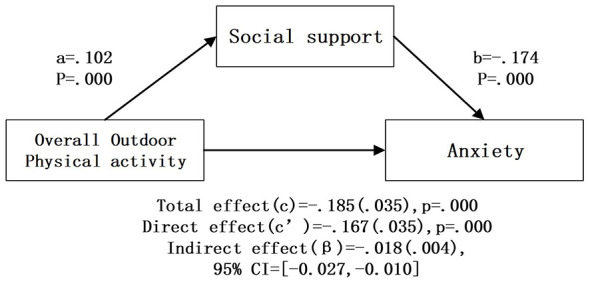
Overall outdoor activityintermediary inspection results.

**Figure 3:**
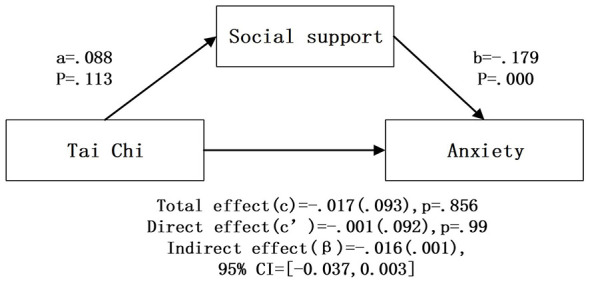
Tai Chi intermediary inspection results.

**Figure 4:**
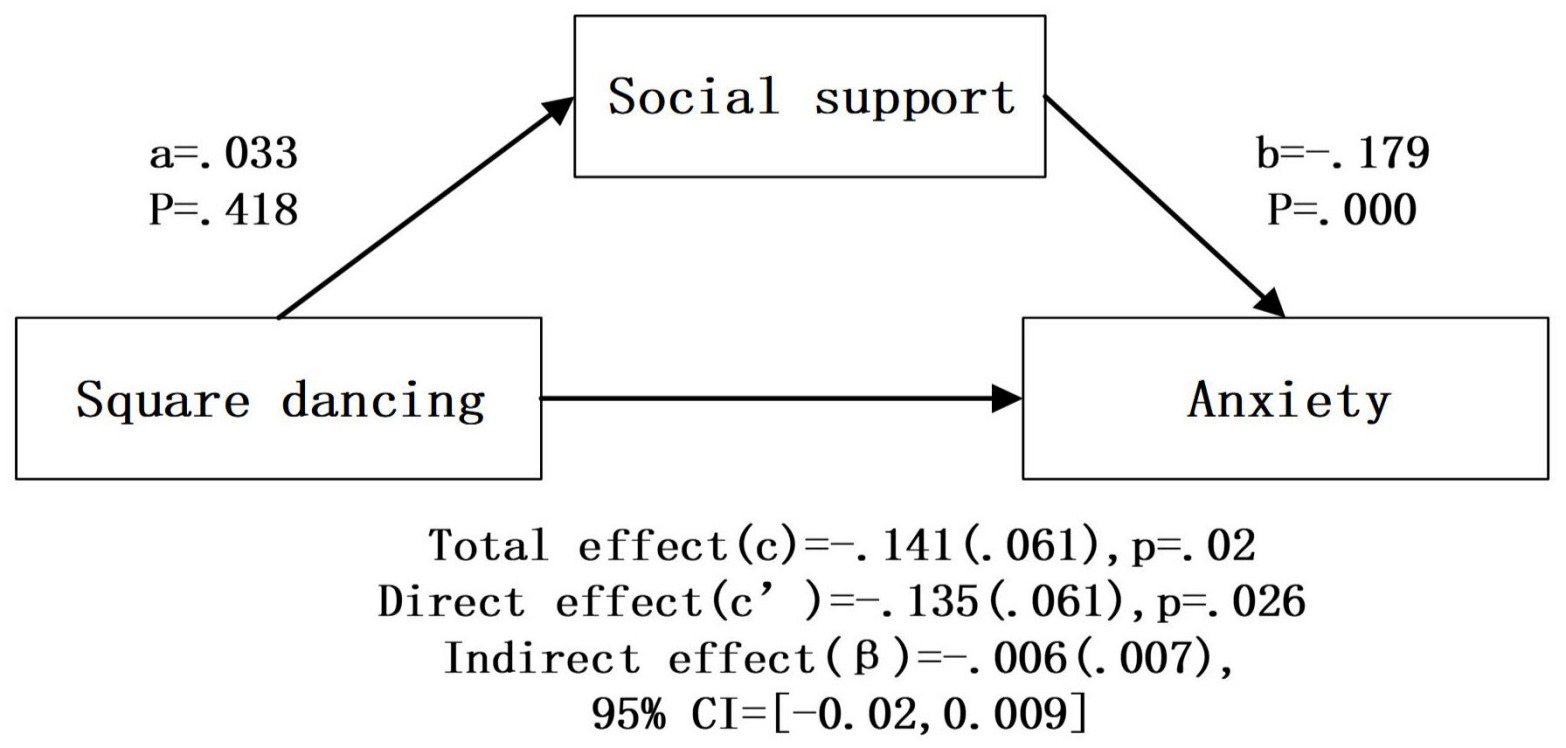
Square dancing intermediary inspection results.

**Figure 5:**
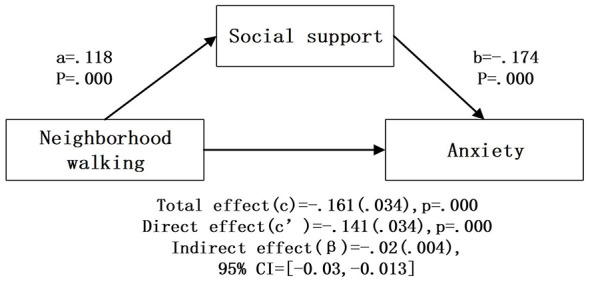
Neighborhood walking intermediary inspection results.

**Figure 6:**
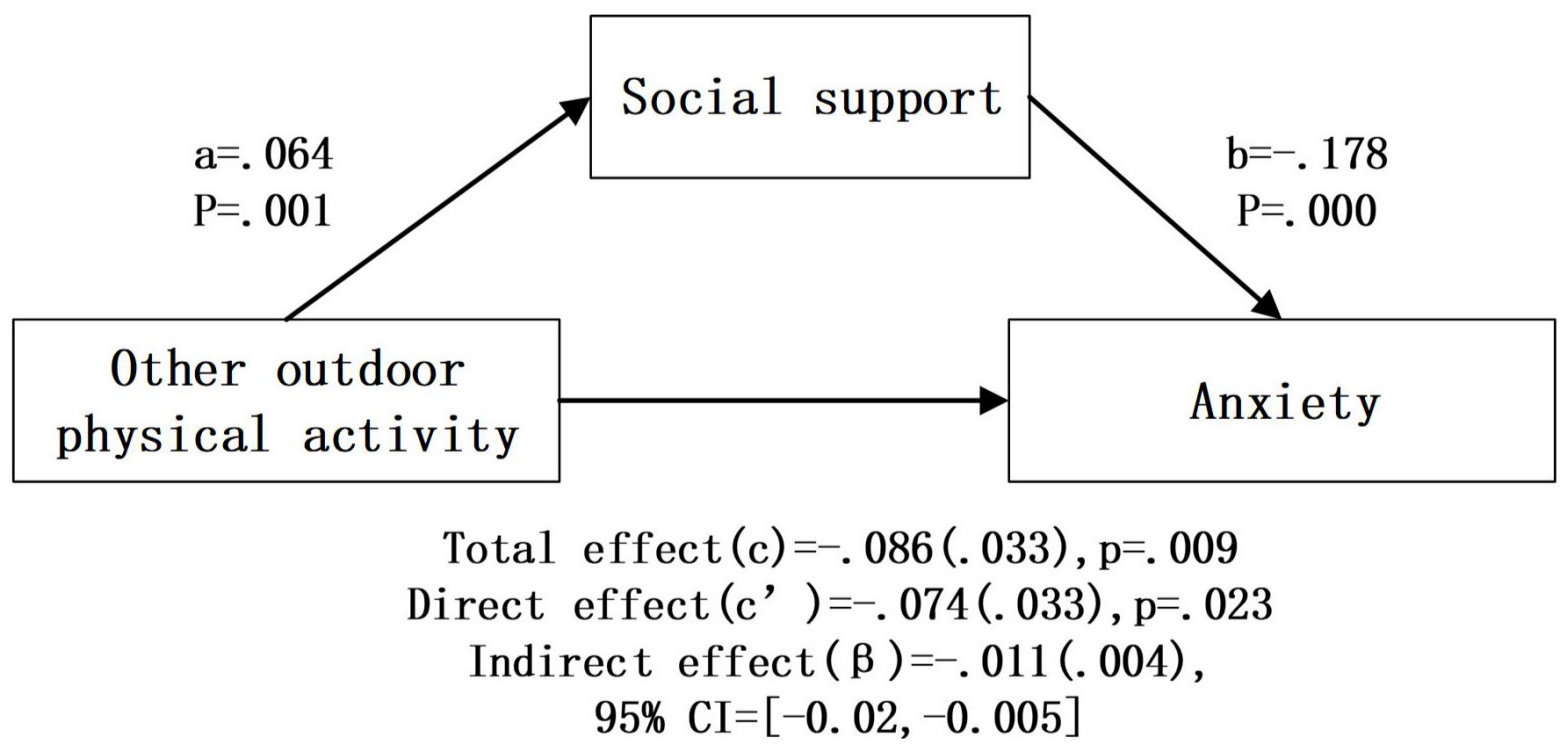
Other outdoor activity intermediary inspection results.

After controlling for covariates, bootstrap mediation analysis (Table 4; Figures 2–6) showed that higher social support scores were statistically associated with lower anxiety scores across all models (b = −0.174 to −0.179, *p* < 0.001). Overall outdoor activity frequency significantly and positively predicted social support (a = 0.102, *p* < 0.001), and social support in turn significantly and negatively predicted anxiety (b = −0.174, *p* < 0.001). The direct effect of overall outdoor activity frequency on anxiety was also significant (c′= −0.167, *p* < 0.001). Neighborhood walking had a significant indirect effect on anxiety through social support [β = −0.020 (0.004)], 95% CI [−0.030, −0.013]. Neighborhood walking significantly and positively predicted social support (a = 0.118, *p* < 0.001), and social support significantly and negatively predicted anxiety (b = −0.174, *p* < 0.001); the direct effect of neighborhood walking on anxiety was also significant (c′= −0.141, *p* < 0.001). Other outdoor activities also showed a significant indirect effect through social support [β = −0.011 (0.004)], 95% CI [−0.020, −0.005]. Other outdoor activities significantly and positively predicted social support (a = 0.064, *p* = 0.001), and social support significantly and negatively predicted anxiety (b = −0.178, *p* < 0.001); the direct effect was also significant (c′= −0.074, *p* = 0.023). In addition, overall outdoor activity frequency showed a significant indirect effect through social support [β = −0.018 (0.004)], 95% CI [−0.027, −0.010].

**Table 4:** Analysis of the mediating effects of outdoor activities.

Variable	Effect type	Coef	SE	Bootstrap 95% CI	*P*	Effect proportion (%)
**Tai Chi**	a	0.088	0.055	[−0.021, 0.192]	0.113	
	b	**−0.179**	**0.024**	**[−0.225**, **−0.133]**	**<0.001** ^***^	
	Indirect effect (a × b)	−0.016	0.01	[−0.037, 0.003]	0.122	94.12
	Direct effect (c′)	−0.001	0.092	[−0.175, 0.187]	0.99	5.88
	Total effect (c)	−0.017	0.093	[−0.189, 0.176]	0.856	
**Square dancing**	a	0.033	0.041	[−0.055, 0.108]	0.418	
	b	**−0.179**	**0.024**	**[−0.228**, **−0.132]**	**<0.001** ^***^	
	Indirect effect (a × b)	−0.006	0.007	[−0.020, 0.009]	0.425	4.26
	Direct effect (c′)	**−0.135**	**0.061**	**[−0.246**, **−0.008]**	**0.026** ^*^	95.74
	Total effect (c)	**−0.141**	**0.061**	**[−0.251**, **−0.012]**	**0.02** ^*^	
**Neighborhood walking**	a	0.118	**0.019**	**[0.082, 0.154]**	**<0.001** ^***^	
	b	−0.174	**0.024**	**[−0.222**, **−0.127]**	**<0.001** ^***^	
	Indirect effect (a × b)	**−0.02**	**0.004**	**[−0.030**, **−0.013]**	**<0.001** ^***^	12.42
	Direct effect (c′)	**−0.141**	**0.034**	**[−0.210**, **−0.075]**	**<0.001** ^***^	87.58
	Total effect (c)	**−0.161**	**0.034**	**[−0.229**, **−0.095]**	**<0.001** ^***^	
**Other outdoor activities**	a	0.064	**0.019**	**[0.026, 0.102]**	**0.001** ^**^	
	b	**−0.178**	**0.024**	**[−0.226**, **−0.131]**	**<0.001** ^***^	
	Indirect effect (a × b)	**−0.011**	**0.004**	**[−0.020**, **−0.005]**	**0.003** ^**^	12.79
	Direct effect (c′)	**−0.074**	**0.033**	**[−0.137**, **−0.011]**	**0.023** ^*^	86.05
	Total effect (c)	**−0.086**	**0.033**	**[−0.149**, **−0.022]**	**0.009** ^**^	
**Overall outdoor activity**	a	**0.102**	**0.019**	**[0.064, 0.141]**	**<0.001** ^***^	
	b	**−0.174**	**0.024**	**[−0.220**, **−0.127]**	**<0.001** ^***^	
	Indirect effect (a × b)	**−0.018**	**0.004**	**[−0.027**, **−0.010]**	**<0.001** ^***^	9.73
	Direct effect (c′)	**−0.167**	**0.035**	**[−0.236**, **−0.100]**	**<0.001** ^***^	90.27
	Total effect (c)	**−0.185**	**0.035**	**[−0.255**, **−0.117]**	**<0.001** ^***^	

^***^p < 0.001, ^**^p < 0.01, ^*^p < 0.05.

In contrast, Tai Chi showed no significant indirect effect and did not significantly predict social support, although social support remained a significant negative predictor of anxiety (b = −0.179, *p* < 0.001). Similarly, square dancing had a significant direct effect on anxiety (c′= −0.135, *p* = 0.026) whereas the indirect path through social support was not significant; at the same time, higher social support scores remained statistically associated with lower anxiety scores (b = −0.179, *p* < 0.001).

In subgroup mediation analyses (Table 5), significant indirect effects through social support were observed for other outdoor activity, overall outdoor activity, and neighborhood walking. For other outdoor activity, significant indirect effects were found in participants aged ≥90 years [β = −0.020 (0.008)], 95% CI [−0.035, −0.005], those with chronic conditions [β = −0.014 (0.005)], 95% CI [−0.024, −0.004], town residents [β = −0.016 (0.008)], 95% CI [−0.031, −0.001], and women [β = −0.017 (0.007)], 95% CI [−0.031, −0.004]. For overall outdoor activity, significant indirect effects were observed in participants aged ≥90 years [β = −0.021 (0.007)], 95% CI [−0.036, −0.007], those with chronic conditions [β = −0.021 (0.006)], 95% CI [−0.032, −0.010], city residents [β = −0.018 (0.008)], 95% CI [−0.034, −0.002], town residents [β = −0.027 (0.010)], 95% CI [−0.046, −0.008], women [β = −0.023 (0.007)], 95% CI [−0.036, −0.010], and men [β = −0.013 (0.005)], 95% CI [−0.023, −0.003]. For neighborhood walking, significant indirect effects were found in participants aged 80–89 years [β = −0.015 (0.008)], 95% CI [−0.030, −0.000], those aged ≥90 years [β = −0.028 (0.009)], 95% CI [−0.046, −0.010], those with chronic conditions [β = −0.024 (0.006)], 95% CI [−0.035, −0.013], city residents [β = −0.020 (0.009)], 95% CI [−0.038, −0.002], town residents [β = −0.027 (0.010)], 95% CI [−0.046, −0.008], women [β = −0.027 (0.007)], 95% CI [−0.041, −0.013], and men [β = −0.014 (0.005)], 95% CI [−0.024, −0.003]. Other subgroup results are shown in the Supplementary Tables.

**Table 5:** Significant subgroup-specific indirect effects of outdoor activities on anxiety through social support.

Variable		Category	Coef	SE	Bootstrap 95% CI	*P*
Other outdoor activity	Age	≥90 years	−0.020	0.008	[−0.035, −0.005]	0.009
	Chronic conditions	yes	−0.014	0.005	[−0.024, −0.004]	0.005
	Place of residence	town	−0.016	0.008	[−0.031, −0.001]	0.036
	Sex	female	−0.017	0.007	[−0.031, −0.004]	0.014
Overall outdoor activity	Age	≥90 years	−0.021	0.007	[−0.036, −0.007]	0.004
	Chronic conditions	yes	−0.021	0.006	[−0.032, −0.010]	0.000
	Place of residence	city	−0.018	0.008	[−0.034, −0.002]	0.032
		town	−0.027	0.010	[−0.046, −0.008]	0.005
	Sex	female	−0.023	0.007	[−0.036, −0.010]	0.001
		male	−0.013	0.005	[−0.023, −0.003]	0.014
Neighborhood walking	Age	80–90 years	−0.015	0.008	[−0.030, −0.000]	0.047
		≥90 years	−0.028	0.009	[−0.046, −0.010]	0.002
	Chronic conditions	yes	−0.024	0.006	[−0.035, −0.013]	0.000
	Place of residence	city	−0.020	0.009	[−0.038, −0.002]	0.028
		town	−0.027	0.010	[−0.046, −0.008]	0.005
	Sex	female	−0.027	0.007	[−0.041, −0.013]	0.000
		male	−0.014	0.005	[−0.024, −0.003]	0.009

## 4 Discussion

This study examined the mediating role of social support in the relationship between Tai Chi, square dancing, neighborhood walking, other outdoor activities, and anxiety. It also investigated the combined effects of overall outdoor activity and social support on anxiety by aggregating the frequency of various outdoor activities. Findings indicate that 11.57% of older adults exhibit anxiety susceptibility. Higher susceptibility rates were observed among women, individuals with chronic diseases, town/rural residents, those with primary school education or below, low-income individuals, and those who never engage in outdoor activity, neighborhood walking, or other outdoor activities. The observed correlations were small: social support and overall outdoor activity were only weakly associated with anxiety, and the activity-specific correlations with anxiety were largely non-significant. The mediation results are therefore better viewed as evidence of a limited but detectable pathway than as evidence of a strong explanatory mechanism (36). A similar point applies to the regression models. Although several coefficients reached statistical significance, their magnitudes were modest, suggesting that the practical significance of these associations at the individual level may be limited and that outdoor activity and social support likely capture only part of a broader set of factors related to anxiety in later life. This does not mean that the findings lack relevance. Routine outdoor activities such as neighborhood walking are common, low-cost, and potentially scalable, so even modest coefficients may still carry public health importance when considered at the population level. Such an interpretation is also consistent with prospective studies and review evidence showing that physical activity is associated with lower anxiety risk, while the size of these associations is typically modest and not uniformly significant across different anxiety outcomes (11, 28).

What might lie behind this modest association? In the adjusted mediation model, the link between more frequent outdoor activity and lower anxiety scores coincided with a statistically significant indirect path through social support, which accounted for 9.56% of the total estimated association. One plausible reading is that outdoor activity may be associated with lower levels of anxiety because it is linked to higher social support indirectly via a number of possible pathways. Outdoor activities in most communities are usually socially oriented which means people have opportunities to engage in casual recreation interactions with others thus slowly building relationships and sense of community through these interactions. Regular interaction between members can make them feel more accepted and appreciated by the group, which could help to reduce the response to stress, as well as decrease over-worrying and ruminative thought patterns (32). Regular group outdoor activities can also establish predictable daily routines and informal expectations (37, 38), which may decrease inactive behaviors and withdrawal behavior to reduce the manifestation of anxiety symptoms and improve sleeping problems (39). This effect offers a plausible context for the social-support pattern observed. The model suggests that social relations can impact mental health directly in a beneficial manner as well as buffer against adverse effects of stressors (32, 40).The results showed that about 72.76% of the older adults had chronic illness. Collective physical activity participation, as well as education sessions on managing a chronic condition helped this group learn more about living with a chronic disease, enhance their sense of self-efficacy to control the diseases, increase information sources, and reduce concern (24). Several studies of relationships among older adults' social networks and health indicate that such variables as network size, variety of social roles, frequency of social contact, and perceived sense of support have meaningful long-term effects on mental health outcomes as well as mortality (41–43). With regard to the aspect of social contact it can be stated that spending time outdoors is not only a physical activity but also greater social integration.

Longitudinal research indicates that social disengagement and perceived isolation are significantly and positively associated with depression and anxiety in older adults, and reduced social participation can heighten emotional burden through mediating pathways such as loneliness (26, 44). Using NSHAP data from the United States, a longitudinal mediation analysis further showed that objective social disengagement predicted subsequent increases in perceived isolation, which in turn contributed to higher levels of depressive and anxiety symptoms (26).

Neighborhood walking showed a significant direct effect and the relatively largest significant mediated effect, may suggest a closer association with neighborhood interaction, community embeddedness, and informal social support networks (45, 46). Neighborhood walking is a low-intensity, sustainable daily activity that takes place near one's home, within familiar and semi-familiar social networks. It requires little additional cost or skill and more readily forms a high-frequency, short-duration, repeatable routine of going out. This can increase chance encounters, greetings, and small reciprocal interactions, building familiarity and a sense of belonging in the community over time and ultimately manifesting as higher perceived support and lower anxiety burden (31, 47, 48). Open space and nearby public spaces are thought to support both activity and socializing among older adults, thereby influencing mental health-related outcomes (31). Walkability and better neighborhood social environments has been associated with higher levels of physical activity and social interaction among the aged, that could have an impact on psychological well being outcome (49), comparison with other outdoor activities like playing ball sports bicycling, or swimming, the level of activity necessary to participate in local walks is fairly low: enabling people with reduced mobility to join in. Previous studies show that older adults' walking behavior is highly associated with their ability of mobility, and safe and convenient walking conditions may be key to enabling outings and maintaining social contact (50).

We found that the results for Tai Chi were not statistically significant, and the indirect effect of square dancing through social support was not significant; directionally, square dancing appeared to operate mainly through a direct effect. Multiple randomized controlled trials and systematic reviews have shown that mind–body integrative physical activity such as Tai Chi and Baduanjin can improve older adults' emotional symptoms, sleep, and overall mental health (51). Arts-based interventions, including dance and group music, have been associated with improvements in depression and anxiety among older adults (52, 53). In China, square dancing has become one of the most popular group activities among older women (54). It combines physical activity with musical and aesthetic elements, while also offering consistent opportunities for social connection among peers. It has been shown in studies that exercising like Tai Chi reduces the symptoms of depression (55). However, some results are different than previous studies, such as social support had no significant mediation effect on square dancing. One of the most important features of this sample is that the proportion of people who have not participated in Tai Chi or square dance is very high [97.24% for Tai Chi; 94 non-participants (99%)]. This highly skewed exposure distribution meant that effective comparisons occurred within a very small subgroup, diluting statistical effects. In addition, categorizing activity frequency may not reflect actual dose (intensity, duration, and years of practice), making true effects harder to detect in mediation models. Given the sample's age structure (a high proportion of the oldest-old), those who can continue participating in Tai Chi or square dancing may represent a health-selected group of survivors (56), whereas many individuals with limited physical capacity and functional impairments may rely more on low-intensity walking and other simple outdoor activities. Under these conditions, the marginal effects of daily walking and other outdoor activities may be further amplified (57). These result also suggests that initiatives which aim for increased engagement on mild intensity outdoor social activity may be easier to scale-up amongst the older age cohorts than those which seek to encourage their engagement in leisure time outdoor activities like Tai Chi and square dancing (58).

The effects we found as relationships and mediators in our study are small to moderate; thus, it is possible that overall public health outcomes could be better understood through population-level gains instead of large personal changes for any one person alone. Our results are consistent with current reviews on physical activity and mental health or prospective studies focusing on physical activity and symptoms of anxiety (28). For improve older adult mental health, Policies to improve older adult mental health should combine efforts at creating access to nature-based activity and the creation of opportunities for community communication. To do so requires urban development which makes it easier for the older adults to move about, including walkability improvements, more public space for socializing, and micro-infrastructure projects such as park upgrades, facilities, seating options, lighting, and other features of a venue that may render it more accessible for people with physical disabilities (59, 60). Evidence also suggests that age-friendly community environments can indirectly enhance older adults' mental health and subjective wellbeing by strengthening social capital and increasing physical activity (61). From a social support perspective, community interventions could prioritize neighbor mutual-aid and small-group participation as organizational platforms, and incorporate volunteer services and peer support into routine programming to strengthen social connectedness and access to emotional support (62). Structured intergenerational interaction may serve as a useful supplement: pairing younger volunteers with older adults can extend sources of support beyond the family to the community relationship network, improving access to both emotional support and practical help (63). For example, in regard to policy for aging-in-place, creating such community-based social capital is associated with higher wellbeing outcomes (e.g., life satisfaction, while building a better social infrastructure to support long term care, Without family support, frail older adults people might experience difficulties living at home (64). There are targeted populations that need specific attention such as older women, frail elders, low education and income, and older persons who live in small towns or rural areas: Such groups might especially respond well to individualized interventions for promoting ambulation and socialization, including implementation of modified physical therapy modalities such as the use of lifestyle integrated functional physical activity in outdoor to minimize the incidence of falls (65). The mediation findings suggest that the social context of activity should not be overlooked. Because social support statistically accounted for part of the association between outdoor activity and anxiety, community strategies that strengthen regular social contact may be relevant, especially for older adults who are socially isolated or have limited mobility (26). In this context, volunteer-based companionship or assisted outings may serve as practical ways to expand social contact and perceived support, which is consistent with recent evidence that structured volunteering can reduce loneliness in older adults (62). In health-care settings such as nursing homes and hospitals, support may also depend on the quality of communication. Clear, understandable information and practical guidance from health-care staff may help reduce uncertainty, improve participation in care, and support better patient-centered outcomes (24, 66).These implications should be interpreted as extensions of the social-support pathway suggested by our findings, rather than interventions directly tested in the present study.

The subgroup analyses suggest that the social-support pathway was not equally evident across the sample. Indirect effects appeared more often in the oldest-old, in participants with chronic conditions, and in town or city residents, while the sex differences were present but modest. Given the small overall correlations and modest coefficients in this study, these subgroup patterns should be interpreted cautiously. Even so, they suggest that the association between outdoor activity and anxiety may be more detectable through social support in groups whose daily mobility and social participation are more closely tied to nearby environments and routine interpersonal contact (30). This interpretation may be particularly relevant for very old adults and those living with chronic conditions, for whom everyday support resources are likely to be more salient in daily life (43), and for urban and semi-urban settings, where neighborhood environments may provide more regular opportunities for repeated contact and perceived support (30, 47).

This study has several strengths. It used a large national CLHLS sample of older adults, with broad population coverage and a substantial sample size. It also distinguished different types of outdoor activity rather than relying only on an overall measure of physical activity. Several limitations should also be noted. Because the design was cross-sectional, the temporal ordering and causal direction among outdoor activity frequency, social support, and anxiety could not be established. Participation in Tai Chi and square dancing was low, and the resulting exposure imbalance may have reduced statistical power and increased measurement error. A single composite social support score could not separate structural support from functional support, which may have obscured activity-specific social mechanisms. The CLHLS dataset mainly provided information on participation frequency for different types of outdoor activity. We were therefore unable to account for frequency, duration, intensity, and years of practice, which may have led to exposure misclassification and reduced the interpretability of the findings. Because several observed correlations were small in magnitude, the present models are likely to capture only a limited proportion of the processes linking outdoor activity to anxiety in later life. In addition, although multiple covariates were adjusted for, residual confounding from unmeasured factors cannot be excluded. Unmeasured variables such as psychological vulnerability, cognitive status, mobility limitations, pain severity, sleep problems, medication use, and neighborhood environmental characteristics may have influenced both outdoor activity participation and anxiety, and may contribute to the modest magnitude of the observed associations and the relatively small indirect effects detected in the mediation analysis. Future studies should use longitudinal or intervention designs, more detailed outdoor activity measures, consider additional outcomes such as depression and life satisfaction, and develop multidimensional models of mental health, as well as separate indicators of structural and functional support to strengthen mechanistic inference and practical applicability.

## 5 Conclusion

Using a nationally representative sample of older adults in China, this study examined the associations of Tai Chi, square dancing, neighborhood walking, other outdoor activities, and overall outdoor activity frequency with anxiety, while considering social support as a potential mediator within the same analytic framework. The findings suggest that overall outdoor activity, neighborhood walking, and other outdoor activities were associated with lower anxiety scores, and that these associations were partly linked to social support. By contrast, the indirect effects through social support were not statistically significant for Tai Chi or square dancing. Social support, measured as a composite indicator covering family-based support, caregiving assistance, financial support, children's visits, and access to community services and social insurance, was consistently and negatively associated with anxiety symptoms. Although the observed associations and indirect effects were weak, the results point to the potential public health relevance of routine, accessible outdoor activities embedded in everyday life. In later life, reducing barriers to safe and convenient outdoor participation, while strengthening opportunities for social connection and support, may be relevant to anxiety prevention and healthy aging at the population level.

## Data Availability

Publicly available datasets were analyzed in this study. This data can be found here: https://opendata.pku.edu.cn/.
