# Supplementary material for: Outdoor activities and social support on anxiety in Chinese older adults: a cross-sectional study
Source: Front Public Health. 2026 May 18;14:1817922. doi: 10.3389/fpubh.2026.1817922 (PMC13223094; doi:10.3389/fpubh.2026.1817922)
Supplement: Supplementary file 1 [file Table_1.DOCX]

Supplementary Table S1. Significant subgroup-specific direct, indirect, and total effects of outdoor activities on anxiety through social support

| **Variable** |  | **Category** | **Effect Type** | **Coef** | **SE** | **Bootstrap 95% CI** | **P** |
| --- | --- | --- | --- | --- | --- | --- | --- |
| Overall outdoor activity | Age | 70–<80 years | Direct | -0.257 | 0.088 | [-0.429, -0.085] | 0.003 |
|  |  |  | Total | -0.269 | 0.090 | [-0.446, -0.092] | 0.003 |
|  |  | 80–90 years | Direct | -0.176 | 0.069 | [-0.310, -0.042] | 0.010 |
|  |  |  | Total | -0.189 | 0.068 | [-0.322, -0.055] | 0.006 |
|  |  | <70 years | Direct | -0.360 | 0.136 | [-0.627, -0.093] | 0.008 |
|  |  |  | Total | -0.371 | 0.135 | [-0.637, -0.106] | 0.006 |
|  |  | ≥90 years | Indirect | -0.021 | 0.007 | [-0.036, -0.007] | 0.004 |
|  |  |  | Total | -0.119 | 0.050 | [-0.217, -0.021] | 0.017 |
|  | Chronic conditions | yes | Direct | -0.225 | 0.042 | [-0.308, -0.142] | 0.000 |
|  |  |  | Indirect | -0.021 | 0.006 | [-0.032, -0.010] | 0.000 |
|  |  |  | Total | -0.246 | 0.043 | [-0.330, -0.162] | 0.000 |
|  | Place of residence | city | Indirect | -0.018 | 0.008 | [-0.034, -0.002] | 0.032 |
|  |  | rural | Direct | -0.172 | 0.051 | [-0.271, -0.073] | 0.001 |
|  |  |  | Total | -0.176 | 0.051 | [-0.275, -0.076] | 0.001 |
|  |  | town | Direct | -0.242 | 0.069 | [-0.378, -0.106] | 0.000 |
|  |  |  | Indirect | -0.027 | 0.010 | [-0.046, -0.008] | 0.005 |
|  |  |  | Total | -0.269 | 0.070 | [-0.406, -0.133] | 0.000 |
|  | Sex | female | Direct | -0.156 | 0.051 | [-0.257, -0.056] | 0.002 |
|  |  |  | Indirect | -0.023 | 0.007 | [-0.036, -0.010] | 0.001 |
|  |  |  | Total | -0.179 | 0.051 | [-0.280, -0.079] | 0.000 |
|  |  | male | Direct | -0.195 | 0.047 | [-0.286, -0.104] | 0.000 |
|  |  |  | Indirect | -0.013 | 0.005 | [-0.023, -0.003] | 0.014 |
|  |  |  | Total | -0.207 | 0.047 | [-0.299, -0.116] | 0.000 |
| neighborhood walking | Age | 70–<80 years | Direct | -0.194 | 0.072 | [-0.335, -0.054] | 0.007 |
|  |  |  | Total | -0.202 | 0.073 | [-0.346, -0.059] | 0.006 |
|  |  | 80–90 years | Indirect | -0.015 | 0.008 | [-0.030, -0.000] | 0.047 |
|  |  |  | Total | -0.134 | 0.064 | [-0.259, -0.009] | 0.035 |
|  |  | <70 years | Direct | -0.312 | 0.115 | [-0.537, -0.087] | 0.007 |
|  |  |  | Total | -0.327 | 0.114 | [-0.551, -0.103] | 0.004 |
|  |  | ≥90 years | Indirect | -0.028 | 0.009 | [-0.046, -0.010] | 0.002 |
|  |  |  | Total | -0.117 | 0.051 | [-0.218, -0.017] | 0.023 |
|  | Chronic conditions | yes | Direct | -0.182 | 0.040 | [-0.261, -0.103] | 0.000 |
|  |  |  | Indirect | -0.024 | 0.006 | [-0.035, -0.013] | 0.000 |
|  |  |  | Total | -0.206 | 0.041 | [-0.286, -0.127] | 0.000 |
|  | Place of residence | city | Indirect | -0.020 | 0.009 | [-0.038, -0.002] | 0.028 |
|  |  | rural | Direct | -0.132 | 0.048 | [-0.227, -0.037] | 0.007 |
|  |  |  | Total | -0.141 | 0.049 | [-0.237, -0.046] | 0.004 |
|  |  | town | Direct | -0.213 | 0.064 | [-0.340, -0.087] | 0.001 |
|  |  |  | Indirect | -0.027 | 0.010 | [-0.046, -0.008] | 0.005 |
|  |  |  | Total | -0.240 | 0.065 | [-0.368, -0.113] | 0.000 |
|  | Sex | female | Direct | -0.116 | 0.050 | [-0.214, -0.018] | 0.020 |
|  |  |  | Indirect | -0.027 | 0.007 | [-0.041, -0.013] | 0.000 |
|  |  |  | Total | -0.143 | 0.050 | [-0.241, -0.045] | 0.004 |
|  |  | male | Direct | -0.178 | 0.042 | [-0.261, -0.095] | 0.000 |
|  |  |  | Indirect | -0.014 | 0.005 | [-0.024, -0.003] | 0.009 |
|  |  |  | Total | -0.192 | 0.043 | [-0.276, -0.108] | 0.000 |
| Other outdoor activity | Age | ≥90 years | Indirect | -0.020 | 0.008 | [-0.035, -0.005] | 0.009 |
|  | Chronic conditions | yes | Indirect | -0.014 | 0.005 | [-0.024, -0.004] | 0.005 |
|  |  |  | Total | -0.079 | 0.039 | [-0.157, -0.002] | 0.045 |
|  | Place of residence | town | Indirect | -0.016 | 0.008 | [-0.031, -0.001] | 0.036 |
|  |  |  | Total | -0.131 | 0.064 | [-0.257, -0.005] | 0.042 |
|  | Sex | female | Indirect | -0.017 | 0.007 | [-0.031, -0.004] | 0.014 |
|  |  | male | Direct | -0.116 | 0.041 | [-0.196, -0.037] | 0.004 |
|  |  |  | Total | -0.123 | 0.041 | [-0.203, -0.043] | 0.003 |
